# Childhood body mass index trajectories and risk of overweight and obesity in young adulthood: a population-based prospective cohort study

**DOI:** 10.1007/s00431-026-07080-5

**Published:** 2026-05-21

**Authors:** Jasmin M. de Groot, Janine F. Felix, Romy Gaillard, Vincent W. V. Jaddoe

**Affiliations:** 1 Generation R Study Group, Erasmus MC, University Medical Center Rotterdam, Rotterdam, Netherlands; 2 Department of Pediatrics, Sophia’s Children’s Hospital, Erasmus University Medical Center, Rotterdam, Netherlands

**Keywords:** Pediatric body mass index, Pediatric obesity, Childhood growth, Childhood adiposity

## Abstract

Childhood body mass index (BMI) change is associated with obesity in adulthood but is often investigated in isolated age windows. This study aimed to identify age windows of BMI changes that are associated with overweight, including obesity, in young adulthood. This study was embedded in a population-based, prospective cohort study among 3528 participants born in Rotterdam, the Netherlands, between 2002 and 2006. Data on gestational-age-adjusted birth weight and sex- and age-adjusted BMI (BMI-SDS) was repeatedly collected between 2 and 20 years in a dedicated research center. Associations between childhood BMI-SDS and young adult (from 17 to 21 years of age) BMI-SDS and overweight/obesity were analyzed. Of children with overweight, including obesity, at 2, 6, 10, and 14 years, 32.6%, 54.0%, 57.4%, and 70.3%, respectively, had overweight around 18 years of age. Positive deviations from the expected BMI at any age were positively associated with odds of overweight at 18, with the strongest effect estimate at 6 years (*p*-values < 0.05). Of children in the highest BMI tertile between 2 and 10 years, only those with BMI growth deceleration, defined as a decrease comparable to crossing a major percentile line on a growth chart, between 2 and 6 years did not have increased risk of overweight at 18 years, whereas all other children in the highest BMI tertile did.

*Conclusion*: Optimizing healthy and reversing unhealthy BMI trajectories in early childhood, especially before 6 years, may be a great opportunity for the prevention of overweight and obesity.

**Table:** 

**What is Known:** • *Childhood growth and body mass index (BMI) are strongly related to obesity in adulthood.* • *Rapid weight gain in infancy, early childhood, and adolescence has all been found to be associated with risk of obesity in both childhood and adulthood.*
**What is New:** • *Based on repeated growth data of 3,528 children from a prospective cohort, associations of childhood BMI with young adult BMI are strongest around age 6, compared to ages 2, 10, and 14 years old.* • *The increased risk of adult overweight/obesity in children in the highest BMI tertile was attenuated in those with growth deceleration before 6 years. After this age, regardless of growth velocity, risk remained increased for children in the highest BMI tertile.*

**Supplementary Information:**

The online version contains supplementary material available at 10.1007/s00431-026-07080-5.

## Introduction

Childhood obesity is a major public health problem, globally affecting more than 150 million children [1]. Childhood obesity may lead to short- and long-term individual health complications, with enormous medical and societal impact, including psychological problems, diabetes, fatty liver disease, and increased risks of cardiovascular-related and all-cause mortality [2–6]. It is also a strong risk factor for adult obesity, and the risk of adult obesity appears to originate at least partially in the earliest phase of life [4, 7–11]. Results from longitudinal studies have identified specific weight and body mass index (BMI) growth patterns in childhood associated with obesity in adulthood [7, 10–13]. For example, both high and low birth weight followed by rapid weight gain in infancy are associated with overweight later in life [8, 12, 14]. Other recent retrospective studies have reported that rapid weight gain not only between 2 and 6 years, but also during or shortly after puberty is associated with adult obesity [10, 14, 15]. Collectively, these findings strongly suggest that weight and BMI growth trajectories in childhood are important for risk of adult overweight and obesity. However, there is still no consensus on which childhood period is most influential for this risk, as many studies analyze specific age windows in isolation. Some studies have found the early childhood period most influential, whereas others suggested weight around puberty to be more influential [5, 10, 15, 16]. Although an imperfect measurement tool for fat mass, especially with the new definitions for clinical obesity, sex- and age-adjusted BMI is still the first and most common measure applied in estimating childhood obesity in primary care and population-based settings [17–19]. Identification of the critical age windows for BMI growth is paramount for developing effective strategies for obesity prevention and childhood obesity remission.

Therefore, in a population-based prospective cohort study from fetal life onwards among 3528 subjects, we aimed to identify specific age windows for BMI growth from birth to 14 years that are associated with the odds of overweight and obesity around 18 years. We also assessed whether decelerated BMI growth in children within higher BMI strata was associated with lower odds of overweight and obesity at the age of 18 years, as compared to children in the stable, middle BMI strata.

## Methods

### Study population

This study was embedded within the Generation R Study, a population-based prospective cohort from early fetal life onwards, in Rotterdam, the Netherlands [20]. The Generation R Study is conducted in accordance with the World Medical Association Declaration of Helsinki and has been approved by the Medical Ethical Committee of Erasmus University Medical Center Rotterdam (MEC 198.782/2001/31). Written informed consent was obtained for all participants [20]. Expectant mothers living in Rotterdam with a due date between April 2002 and January 2006 were eligible for participation and included during pregnancy. They were invited for follow-up measurements every 4 years, with the first visit around 6 years of age. For this study, we selected singleton children from the full cohort of 9749 live-born participants who had data on birth weight, gestational age, at least one BMI measurement between ages 2 and 14 years, and a BMI measurement around 18 years, which was defined as young adulthood. This led to a study population of 3528 children (Supplementary Figure S1).

### Birth weight and body mass index assessments

Birth weight (in grams) was obtained from medical records, and sex- and gestational age-adjusted standard deviation scores (SDS) were calculated based on growth references developed by Niklasson et al. [21]. We used height and weight measurements from the ages of 2 (1.9–2.4), 6 (4.8–8.5), 10 (8.5–12.5), 14 (12.6–16.0), and 18 (16.2–21.7) years. Height and weight around age 2 were obtained from community health centers. From 6 years old onwards, height and weight were measured in the Generation R research center until 18 years. We calculated BMI and sex- and age-adjusted SDS for height using R packages *anthro* and *anthroplus* [22–25]. For those older than 19 years at outcome, we calculated BMI-SDS as if they were exactly 19 years old. We used BMI-SDS cutoffs of < 1 SD, ≥ 1 SD, and ≥ 2 SD to categorize low/normal weight, overweight, and obesity, respectively [23]. We created birth weight and BMI-SDS tertiles based on population-specific percentile cutoffs of each follow-up. Further details of the methods for anthropometric data and BMI-SDS calculations are described in Supplementary Text S1.

### Covariates

We constructed a directed acyclic graph to visualize potential confounder relationships for the main associations (Supplementary Figure S2). Parental and pregnancy variables included maternal age at intake, parity, pre-pregnancy BMI, pregnancy smoking, maternal education, partner BMI, and baseline household income. These factors were assessed at intake via questionnaires, aimed to be collected at enrolment. Child factors, including sex and gestational age at birth, were obtained from medical records [20]. Child ethnicity was defined according to the classification of Statistics Netherlands, obtained from questionnaires at intake [26]. For sensitivity analyses, we adjusted for puberty variables consisting of self-reported Tanner stages and first age of menstruation measured in a subset of children (*N* = 2050) [27]. For further details on covariates and puberty factors, we refer to Supplementary Text S1.

### Statistical analyses

First, we calculated summary statistics for our (non-)imputed study population. We performed a non-participation analysis for missing outcome data and calculated Pearson’s correlation coefficients for all BMI variables. We conducted random forest imputations for missing values in covariates, birth weight, and BMI measurements from 2 to 14 years, using the R-based mice package (version 3.16) [27]. We used inverse probability weighting (IPW) to account for selection bias [28–30]. For details of the imputations and IPWs, we refer to Supplementary Texts S2–3. Second, we used Sankey plots to visualize changes in BMI category distributions (low/normal, overweight, and obesity) across childhood for participants who did and did not have overweight or obesity at 2, 6, 10, or 14 years old. Third, to identify critical periods, we used multiple linear and logistic regression models to analyze the independent associations of BMI-SDS at each age on adult BMI-SDS and odds of overweight/obesity at 18 years. We calculated BMI-SDS standardized residuals for each measurement, except birth, from linear regressions of BMI-SDS on the prior BMI-SDS measurements. The residuals obtained from these regressions were used as our independent BMI variable for the analyses, to isolate the effect of BMI at that time point, independent of previous BMI [31]. Basic models were adjusted for IPWs, height-SDS at outcome, child sex, and age (or age difference for BMI-SDS change). Full models were additionally adjusted for confounders (Supplementary Figure S2), which were assessed for multicollinearity with no variance inflation factor–based violations observed. Fourth, we estimated the proportions and corresponding odds ratios of overweight, including obesity, in young adulthood for each combination of BMI tertile at the start and end of each age window (birth to 2 years; 2 to 6 years; 6 to 10 years; and 10 to 14 years), with the stable middle BMI tertile as reference and adjusted for age at outcome, sex, and IPWs. Finally, we assessed the associations of BMI-SDS change across these same age windows with odds of overweight and obesity and conducted analyses stratified for baseline weight/BMI based on tertiles. To analyze effects of BMI-SDS change, we calculated the delta in BMI-SDS between consecutive measurements, which were categorized into three growth velocities (decelerated, <  − 0.67 SD; stable, − 0.67 to 0.67 SD; accelerated, > 0.67 SD) that represent the crossing of a major percentile line on a growth chart, such as from the 2nd to 9th percentile, 9th to 25th, and 25th to 50th [32, 33]. For these analyses, the middle, stable BMI tertile was the reference, and the same basic/confounder models were applied as in previous analyses. We conducted sensitivity analyses of all full models with adjustment for self-reported puberty factors, similar to previous publications [27]. Specifically, models were adjusted for genital/breast development and pubic hair development for the whole subsample (*N* = 2050), and non-stratified analyses were repeated separately for sex. We also ran sensitivity analyses excluding those exceeding 19 years and conducted a complete case analysis for all non-stratified analyses, and observed no differences. All analyses were conducted using R software (version 4.4.1, R Core Team, Austria) [27].

## Results

### Subject characteristics

In our sample, 52.9% were female, 71.4% were of Dutch/European descent, and 22.2% had overweight/obesity in young adulthood (Table 1). Measurements in young adulthood were conducted around the median age of 18.5 years (range, 16.2; 21.7). Complete BMI follow-up data was available for 55.8% (*N* = 1971). In total, 33.0% (*N* = 1163) missed one measurement, whereas 11.2% missed between two and four measurements. The non-imputed subjected characteristics are presented in Supplementary Table S1. Non-participants were more often non-European, male, and of low birth weight, with lower household income and higher parental BMI (Supplementary Table S2).

**Table 1:** Population characteristics

	***N*** = 3528
Parental characteristics	
Maternal age, years	31.2 (4.9)
Multiparous, % (*n*)	41.4 (1459)
Pre-pregnancy maternal BMI, kg/m^2^	22.4 (14.4; 50.2)
Paternal BMI at intake, kg/m^2^	25.2 (3.5)
Maternal smoking during pregnancy, % (*n*)	13.4 (472)
Household income ≥ 2000 €/month,% (*n*)	68.7 (2412)
Maternal higher education, % (*n*)	52.7 (1853)
Child characteristics	
Female, % (*n*)	52.9 (1867)
European/Dutch, % (*n*)	71.4 (2470)
Child growth characteristics	
Gestational age at birth, weeks	39.9 (25.9; 43.4)
Birth weight, kg	3.44 (0.55)
Birth weight, SDS	− 0.02 (− 4.90; 5.92)
2 years	
Age, years	2.1 (1.9; 2.4)
Body mass index, kg/m^2^	16.5 (1.4)
Body mass index, SDS	0.53 (− 4.36; 6.68)
6 years	
Age, years	6.0 (4.8; 8.5)
Body mass index, kg/m^2^	15.7 (10.7; 38.6)
Body mass index, SDS	0.30 (− 3.24; 6.08)
10 years	
Age, years	9.7 (8.5; 12.5)
Body mass index, kg/m^2^	16.9 (9.7; 42.9)
Body mass index, SDS	0.23 (− 3.63; 4.58)
14 years	
Age, years	13.5 (12.6; 16.0)
Body mass index, kg/m^2^	19.1 (11.0; 50.7)
Body mass index, SDS	0.02 (− 4.23; 4.25)
18 years	
Age, years	18.5 (16.2; 21.7)
Body mass index, kg/m^2^	22.0 (13.7; 49.5)
Body mass index, SDS	0.14 (− 4.26; 5.36)
Underweight, % (*n*)	12.3 (435)
Healthy weight, % (*n*)	65.5 (2310)
Overweight, % (*n*)	15.1 (531)
Obesity, % (*n*)	7.1 (252)

The presented values are the average values observed across all imputed datasets and represent means (SD), medians (range), or valid % (*n*) unless otherwise stated. The mean and standard deviation are given for all normally distributed continuous variables, and the median and range for non-normally distributed variables. All child ages are reported as medians (range), regardless of their distribution

*BMI* body mass index, *SDS* standard deviation score (sex and age adjusted)

### Childhood body mass index

**Fig. 1:**
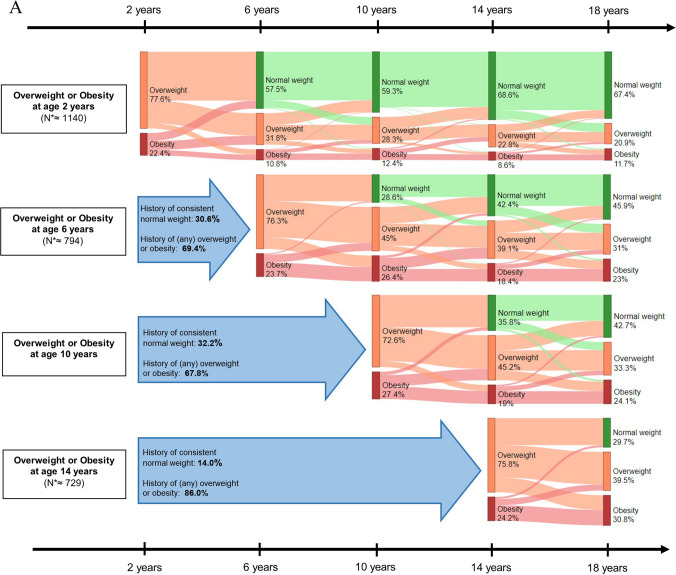

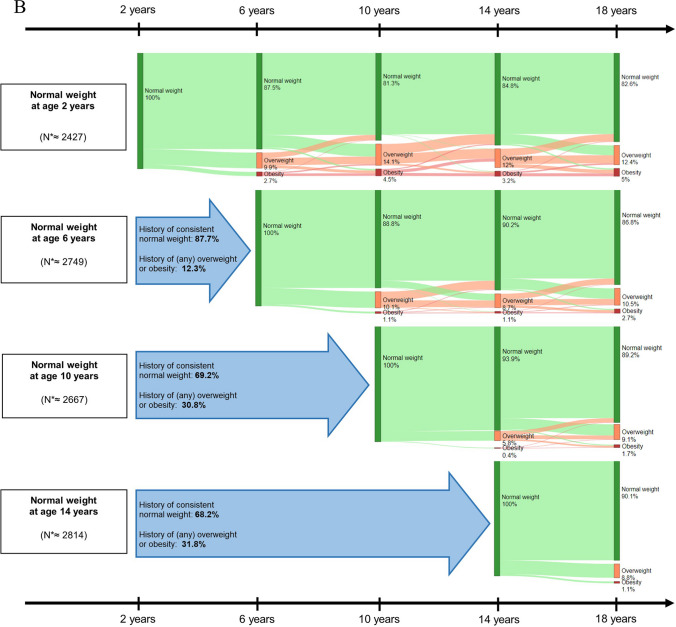
**A **Body mass index trajectories for children with overweight, including obesity, at baseline measurement. The percentages represent the average proportion across the 30 imputed datasets of that weight category per age for children that had overweight, including obesity, at the start of each measurement age in childhood. The arrows before the start of each Sankey plot show the percentage of children who did or did not have a history of overweight, including obesity. *N are based on the average number of observations across all 30 imputed datasets. Abbreviations: SGA, small for gestational age; AGA, appropriate for gestational age; LGA, large for gestational age. Normal weight, overweight, and obesity were defined as < 1 SD, between ≥ 1 SD and < 2 SD, and ≥ 2 SD, respectively. **B **Body mass index trajectories for children with low or normal weight at baseline measurement. The percentages represent the average proportion across the 30 imputed datasets of that weight category per age for children who had a normal weight at the start of each measurement age in childhood. The arrows before the start of each Sankey plot show the percentage of children who did or did not have a history of overweight or obesity. *N are based on the average number of observations across all 31 imputed datasets. Abbreviations: SGA, small for gestational age; AGA, appropriate for gestational age; LGA, large for gestational age. Normal weight, overweight, and obesity were defined as < 1 SD, between ≥ 1 SD and < 2 SD, and ≥ 2 SD, respectively

BMI correlations were weakest between 2 and 18 years (*r* = 0.29) and strongest between 10 and 14 years (*r* = 0.84) (Supplementary Table S3). Of all children with overweight/obesity at ages 2, 6, 10, and 14 years, 32.6%, 54.0%, 57.4%, and 70.3%, respectively, had overweight/obesity at 18 years, and > 66% of these children had a recurring history of overweight (Fig. 1A). In contrast, of children with low/normal BMI at 2, 6, 10, and 14 years, only 17.3%, 13.2%, 10.0%, and 9.9%, respectively, had overweight/obesity at 18 years (Fig. 1B).

**Fig. 2:**
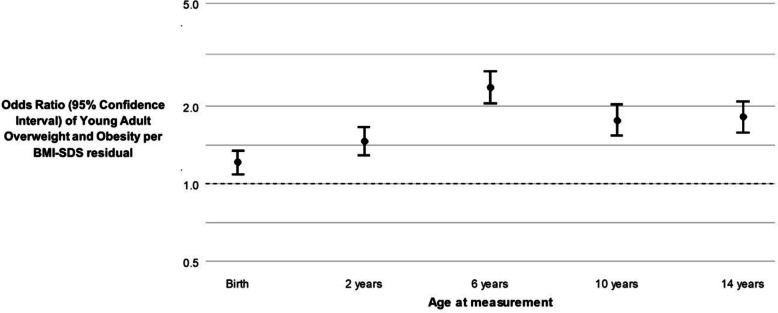
Independent associations of body mass index across childhood with overweight, including obesity, in young adulthood. All odds ratios (OR) and corresponding confidence intervals are pooled standardized estimates of 30 imputed datasets. All *p*-values were FDR-corrected, and all associations survived the multiple testing correction. Only for birth weight did we use the actual SDS as exposure instead of residuals, as there were no previous measurements to regress on. For a complete overview of the effect estimates, we refer to Supplementary Tables S4 and S5

Figure 2 shows that BMI at any age was, independently of BMI at other ages, positively associated with overweight and obesity around 18 years. The residualized BMI-SDS used for these analyses should be interpreted as the deviation of BMI-SDS at measurement age from expected, conditional on their previous BMI-SDS. The strongest association was observed for BMI at 6 years (standardized odds ratio (sOR) 2.36 (95% confidence interval (CI), 2.04; 2.72) per residualized BMI-SDS at 6 years), and weakest for birth weight (sOR 1.20 (95% CI, 1.08; 1.33) per birth weight SD). Corresponding sORs and effect estimates for overweight/obesity and BMI-SDS in young adulthood, respectively, are given in Supplementary Tables S4–5. Sensitivity analyses adjusting for puberty showed similar findings, but with a slightly higher effect estimate for BMI at 14 years (sOR 2.13 (95% CI, 1.81; 2.51)), which was more pronounced in boys (sOR 2.32 (95% CI, 1.80; 2.97)) than girls (sOR 1.82 (95% CI, 1.47; 2.26)) (Supplementary Tables S6–7). Complete case estimates are given in Supplementary Table S8.

### Body mass index changes per age window

**Fig. 3:**
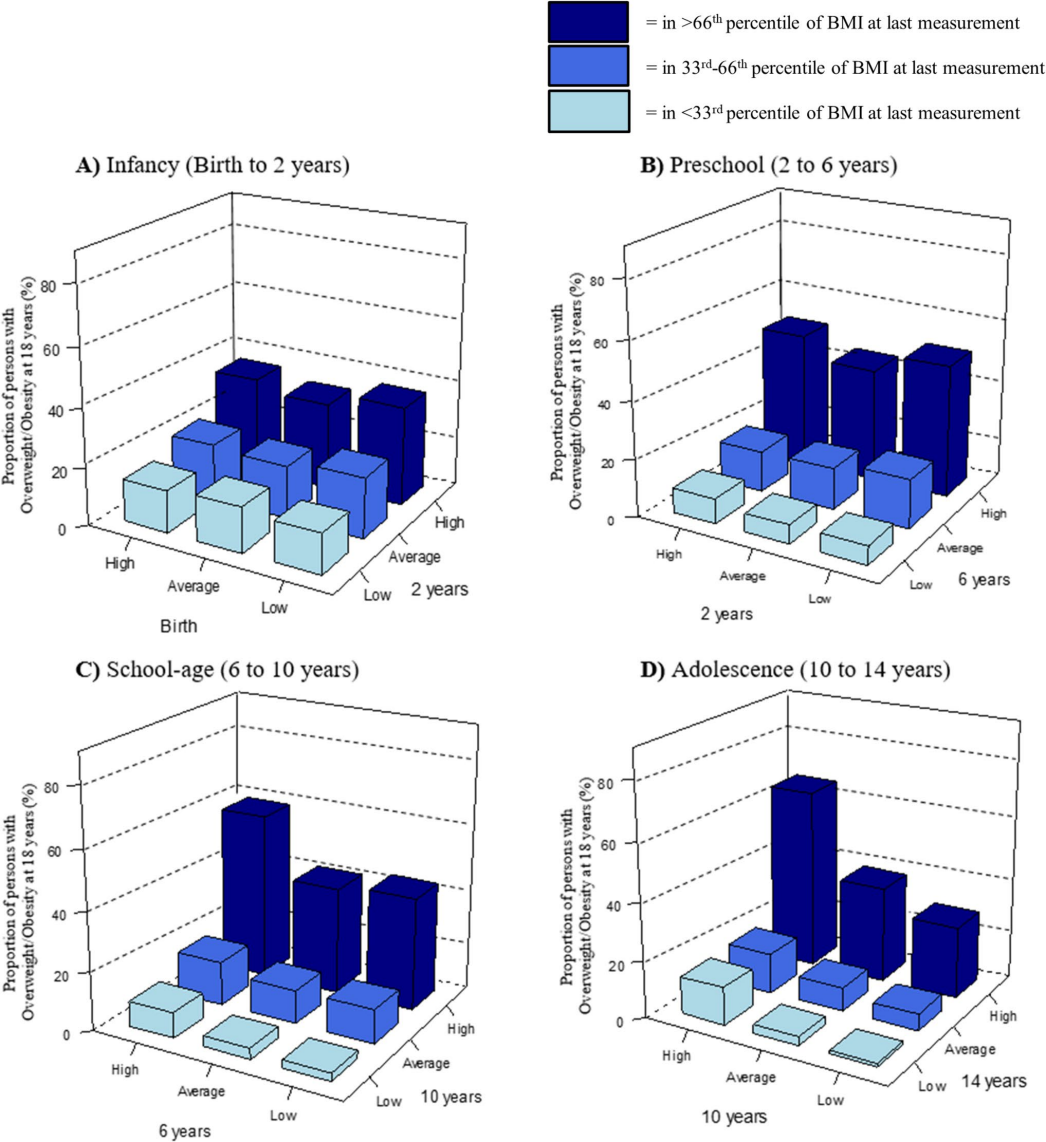
Body mass index change in different age windows and overweight, including obesity, in young adulthood. Each figure represents an age range of change and shows the (pooled) percentage of children with overweight, including obesity, at 18 within each of the 9 different groups of weight change: Each participant starts either at a low, normal, or high BMI category at the first follow-up measurement of the age range, and ends up within a low, normal, or high weight category at the next follow-up measurement of this age range. Low weight was defined as the ≤ 33rd percentile of weight for that age, average weight as between the 33rd and 66th percentiles of weight for that age, and high weight as the ≥ 66th percentile of weight for that age. These percentages are based on the average per imputed dataset (30 imputed datasets). For exact *n*, percentages, and standard errors, see Supplementary Tables S9 and 10

Figure 3A shows that children in the lowest and highest birth weight tertile, followed by a BMI in the highest tertile at age 2 years, had the highest proportions of overweight, including obesity, at 18 years (34.0% (standard error (SE) 2.8) and 33.1% (SE 2.1), respectively). Between 2 and 6 years, the highest percentage of overweight/obesity at 18 years was among children within the lowest or highest BMI tertile who ended up in the highest BMI tertile at 6 years (45.8% (SE 4.1) and 46.4% (SE 1.9), respectively) (Fig. 3B). Between 6 and 10 years and 10 and 14 years, children in the highest BMI tertiles at the start and end of each window (persistently high BMI) had the highest proportions of overweight/obesity at 18 years (56.2% (SE 1.7) and 62.5% (SE 1.6), respectively) (Fig. 3C,D). Corresponding group sizes, percentages, and odds ratios for Fig. 3 are presented in Supplementary Tables S9–10.

Table 2 shows the associations of childhood BMI-growthvelocity with young adult overweight, stratified for weight/BMI tertile. Children in the middle and high birth weight tertiles with accelerated BMI growth until 2 years had increased odds of overweight (*p*-values < 0.05). Accelerated BMI growth between 2 and 6 years or 6 and 10 years was, independently of BMI tertile, associated with increased odds of overweight and obesity (*p*-values < 0.05) (Table 2). From age 10 onwards, only those in the middle and highest BMI tertile with accelerated growth had increased odds of overweight and obesity. Furthermore, children in the highest BMI tertile from age 2 onwards always had a higher likelihood of young adult overweight compared to middle BMI tertile peers, and the size of this likelihood increased with growth velocity. An exception to this finding was those with BMI growth deceleration from 2 to 6 years, whose risk remained similar to that of average BMI peers. The highest odds of overweight were observed for children in the highest BMI tertile at 10 years with accelerated growth (OR 23.67 (95% CI, 9.44; 59.35)) (Table 2). The non-stratified associations of change in BMI are shown in Supplementary Tables S11–12. Adjustment for puberty factors in the non-stratified sensitivity analyses showed differences between sexes: BMI change in boys between birth and 2 years (sOR 1.34 (95% CI, 1.01; 1.79)) and 10 to 14 years (sOR 1.59 (95% CI, 1.26; 2.01)) had a stronger association with young adulthood overweight than girls (sOR 1.06 (95% CI, 0.85; 1.31) and 1.22 (95% CI, 1.02; 1.46), respectively) (Supplementary Table S13–14). Results of the complete case analyses can be found in Supplementary Table S15.

**Table 2:** Change in body mass index per age window and odds of overweight, including obesity, at 18 years

Overweight, including obesity, at age 18 yearsOdds ratio (95% confidence interval)												
Baseline BMI	Birth to 2 years			2 to 6 years			6 to 10 years			10 to 14 years		
	Decelerated	Stable	Accelerated	Decelerated	Stable	Accelerated	Decelerated	Stable	Accelerated	Decelerated	Stable	Accelerated
Lowest tertile	0.99 (0.26; 3.81) *N* = 34	0.63 (0.36; 1.10) *N* = 272	1.35 (0.93; 1.95) *N* = 843	0.32 (0.12; 0.89)* *N* = 101	0.47 (0.31; 0.71)^ǂ^ *N* = 679	1.64 (1.10; 2.45)* *N* = 375	N.A (no cases)^a^ *N* = 138	0.27 (0.17; 0.42)^ǂ^ *N* = 847	1.73 (1.06; 2.81)* *N* = 191	N.A (no cases)^a^ *N* = 185	0.23 (0.13; 0.42)^ǂ^ *N* = 822	0.94 (0.48; 1.82) *N* = 159
Middle tertile	0.80 (0.36; 1.76) *N* = 121	Reference *N* = 488	1.58 (1.06; 2.37)* *N* = 542	0.32 (0.16; 0.65)* *N* = 277	Reference *N* = 718	4.15 (2.37; 7.25)^ǂ^ *N* = 143	0.32 (0.11; 0.92)* *N* = 187	Reference *N* = 804	3.40 (2.12; 5.46)^ǂ^ *N* = 155	0.42 (0.17; 1.05) *N* = 226	Reference *N* = 854	5.45 (2.80; 10.61)^ǂ^ *N* = 82
Highest tertile	1.29 (0.84; 1.94) *N* = 417	1.44 (0.97; 2.12) *N* = 557	2.54 (1.51; 4.26)^ǂ^ *N* = 213	1.10 (0.75; 1.62) *N* = 621	2.86 (2.04; 4.02)^ǂ^ *N* = 488	13.01 (5.81; 29.16)^ǂ^ *N* = 71	2.16 (1.38; 3.38)* *N* = 235	4.41 (3.31; 5.89)^ǂ^ *N* = 826	9.81 (5.51; 17.48)^ǂ^ *N* = 118	2.62 (1.78; 3.88)^ǂ^ *N* = 370	9.66 (7.14; 13.06)^ǂ^ *N* = 751	23.70 (9.50; 59.15)^ǂ^ *N* = 57

All odds ratios (OR) and 95% confidence intervals are pooled estimates taken from 30 imputed datasets. The models were adjusted for age difference, child sex, maternal education and age, parental pre-pregnancy BMI, parity, household income, pregnancy smoking, average sleep at 14, and average screen time at 14. Decelerated and accelerated growth were defined as BMI changes of <  − 0.67 SDS and > 0.67 SDS, respectively. The respective *N* for each group is given with each OR and is based on the average *N* per group across all 31 imputed datasets, rounded to the nearest integer

^*^FDR-adjusted *p* < 0.05

^ǂ^FDR-adjusted *p* < 0.001

^a^This was defined as less than 1 case on average per imputed dataset

## Discussion

We observed that birth weight and positive deviations from expected childhood BMI at any age are, independent of prior BMI, positively associated with overweight, including obesity, in young adulthood. This association was strongest for relative BMI at the age of 6 years and weakest for birth weight. Furthermore, we investigated associations of BMI-SDS growth change equal to crossing a major growth chart percentile line. Of all children with high BMI at 2, 6, or 10 years, only those at 2 years with BMI growth deceleration between 2 and 6 years did not have increased risk of overweight, including obesity, at 18 years, compared to their stable average BMI peers. All other children in the highest BMI stratum had an increased risk of overweight at 18 years, independent of their subsequent growth pattern. Finally, sensitivity analyses suggest that there may be differences between sexes, independent of puberty status.

It is well-established that early childhood BMI and BMI growth are strongly related to the risk of obesity and the associated cardio-metabolic risks in adulthood [3, 8, 11, 34, 37]. Specifically, results from previous studies report that children born large for gestational age or experiencing rapid growth in infancy have an increased risk of obesity and adverse cardio-metabolic risk profiles in later life [5, 35, 36, 38]. Simultaneously, rapid BMI growth around puberty has been found to be associated with adult obesity, where half of adolescents with obesity went on to have adult obesity [15, 39]. In this study, the associations of birth weight, childhood BMI and childhood BMI-growth velocity with risk of overweight and obesity in young adulthood were assessed. For childhood BMI, we observed measurements across the entirety of childhood and found not only relative birth weight but also relative BMI at 2, 6, 10, and 14 years were associated with overweight and obesity in young adulthood. In our sample, children with high BMI-SDS later in childhood were also more likely to have young adult overweight or obesity: a third of children with overweight at 2 years still had overweight in young adulthood, whereas this prevalence was nearly 75% for those with overweight at 14 years. When analyzing BMI at each age independently, BMI at age 6 years showed the strongest association with adult overweight and obesity. These findings suggest that BMI in early childhood may have predictive value, while also indicating substantial potential for overweight remission at younger ages. The particularly strong association observed at age 6 may reflect a critical period, potentially related to adiposity rebound (AR), after which the persistence of overweight becomes more likely. AR is the second rise in body mass index that occurs between 3 and 7 years, and studies have found that an early AR, generally before the age of 5, is a strong risk factor for later obesity [40–43]. It is thought that this timing aids in identifying children whose relative BMI is higher or crossing into higher percentiles which tends to persist in (young) adulthood [44].

For BMI growth, we generally observed that accelerated BMI growth after age 2 years appeared to increase the likelihood of overweight or obesity in young adulthood, regardless of childhood BMI tertile. An exception to this finding were those in the lowest BMI tertile after the age of 10, possibly due to puberty-related growth. However, it should be noted for the interpretation of these findings that acceleration in BMI growth translates to a different absolute BMI change depending on their BMI category/tertile. When we took a closer look at specific BMI tertiles, we observed a nearly consistent, increased likelihood of young adult overweight in children in the highest BMI tertile regardless of growth velocity, except for those children with decelerated growth before 6 years. In line with previous cohort studies, this likelihood appeared to halve with each step down in BMI growth velocity (from accelerated to stable to decelerated), and differences in likelihood became more pronounced with age [15, 39]. One such study among 2732 North American children reported that rates of change in BMI were associated with adult obesity and appeared most crucial between 12 and 15 years and right after 15 years, but weight category appeared most crucial in early childhood [15]. These age windows coincide with puberty developments, and current literature suggests puberty timing and obesity are linked: increased childhood BMI is associated with earlier pubertal timing, especially in girls, but earlier pubertal timing also appears associated with higher BMI later in life, independently of childhood BMI [45, 46]. Our sensitivity analyses with puberty factors showed limited changes in the likelihood of young adult overweight and obesity, although the sample size for the stratified analyses was lacking for some BMI categories. We did, however, observe differences between sexes: growth between birth to 2 years and 10 to 14 years appeared more strongly associated with young adulthood overweight and obesity in boys than in girls, perhaps due to girls generally starting puberty at a younger age or due to the smaller subsample of boys with puberty data [49]. Collectively, our findings suggest that BMI growth velocity may be important to monitor from school-age and perhaps more closely in boys.

For childhood obesity, it is equally relevant to assess potential remission from an unhealthy weight status. As mentioned previously, participants with relatively higher BMI in childhood generally had a higher risk for adult overweight, except for those with decelerated BMI growth before the age of 6: their odds were similar to those in the stable, middle BMI tertile group. Other studies observed similar findings, not limited to just obesity risk: One study showed adverse effects of childhood overweight at age 7 on risk of type 2 diabetes could be reduced by remission of overweight before puberty or maintenance of normal weight until early adulthood [5, 38]. However, the adverse effects of overweight/obesity at age 7 and 13 years were only partly reversible [5]. Our findings from analyses of BMI tertile shifts (Fig. 3) suggest that remission of overweight within a 4-year period from adolescence onwards may be less likely to occur, which could translate to lower impact on young adult BMI-related health outcomes as compared to earlier remission. From an obesity prevention strategy perspective, it therefore seems important to achieve or return to a healthy weight from early childhood onwards [38]. This is not to imply that decelerated BMI growth in children with overweight after early childhood may not have health benefits. On the contrary, our stratified analyses, albeit observational, suggest that the likelihood of young adult overweight is decreased in children with decelerated BMI growth, regardless of when this occurs. However, plasticity of growth and potential for adopting healthy lifestyle habits in early childhood might provide greater opportunities for overweight remission. Furthermore, previous research, primarily in adults, suggests that cardio-metabolic risk associated with obesity is cumulative [16, 47, 48]. A cohort study found overweight from age 13 years to early adulthood was associated with a higher risk of type 2 diabetes compared to later development of overweight [5]. Simultaneously, other studies suggest that remission of obesity before 14 years attenuates the risk of hypertension, type 2 diabetes, and obesity in adulthood [5, 9, 49]. Differences in health effects between children with long-term high BMI, those who remit before adulthood, and those who develop high BMI in (young) adulthood should be further analyzed in longitudinal studies, especially considering the developments in defining (pre)clinical obesity [17]. This new definition highlights the limitations of BMI and the need for future studies to account for body composition and cardio-metabolic risk factors through other measures besides BMI, such as the waist-for-height ratio [17, 18].

Major strengths of this study were the design with repeated BMI data and information on relevant physical, social, and self-reported puberty factors. Most of our sample had data at all or all but one of the follow-ups, allowing us to accurately impute and assess segments of childhood, which previous studies were not always able to. Our study also has limitations. Our sample was a relatively healthy and predominantly European sample of the population of Rotterdam, limiting generalizability. Because of our observational design, residual confounding could not be fully excluded. The number of participants with decelerated/accelerated growth in later childhood was relatively low, partially due to loss to follow-up, and we did not have sufficient data to distinguish between preclinical and clinical obesity [17]. We attempted to attenuate any bias this loss may have introduced through imputation and weighting, but acknowledge this might not have addressed all bias. The study was conducted in a single center and in a single birth cohort, and factors such as the decade, country, and environment in which children grew may have influenced the associations. Ideally, future research should pool data from different cohorts to allow for the exploration of heterogeneity across regions, generations, and countries. Finally, our follow-up was limited to 18–20 years, and further studies are needed to assess the long-term effects on different cardiovascular health outcomes.

## Conclusion

Children with early childhood healthy BMI growth or high BMI followed by a deceleration in BMI growth before the age of 6 years had the lowest likelihood of overweight and obesity in young adulthood. Optimizing and achieving remission of unhealthy BMI trajectories earlier in childhood may be a great opportunity for the prevention of overweight and obesity in young adulthood.

## Supplementary Information

Below is the link to the electronic supplementary material.

ESM 1 (DOCX 129 KB)

## Data Availability

Data used in these analyses are available upon reasonable request from the corresponding author. Proposals should be directed to v.jaddoe@erasmusmc.nl. To gain access, data requestors will need to sign a data access agreement an provide a research proposal for review. If the request is approved, this data consists of deidentified participant data, study protocol, and analytic code. The analytic code is available on GitHub [https://github.com/JMDeGroot/BMI_Change_Manuscript].

## Abbreviations

BMI: Body mass index
IPW: Inverse probability weight
SDS: Standard deviation score
