# Supplementary material for: Childhood body mass index trajectories and risk of overweight and obesity in young adulthood: a population-based prospective cohort study
Source: Eur J Pediatr. 2026 May 21;185(6):427. doi: 10.1007/s00431-026-07080-5 (PMC13194262; doi:10.1007/s00431-026-07080-5)
Supplement: Supplementary file 1 — (DOCX 129 KB) [file 431_2026_7080_MOESM1_ESM.docx]

**Supplementary Files**

**Childhood Body Mass Index Trajectories and Risk of**

**Overweight and Obesity in Young Adulthood:**

**a Population-Based Prospective Cohort Study**

European Journal of Pediatrics

Jasmin M. de Groot, BSc^1,2^, Janine F. Felix, MD, PhD^1,2^, Romy Gaillard, MD, PhD^1,2^, Vincent W.V. Jaddoe, MD, PhD^1,2^

1. Generation R Study Group, Erasmus MC, University Medical Center Rotterdam, Rotterdam, the Netherlands
2. Department of Pediatrics, Erasmus MC, University Medical Center Rotterdam, Rotterdam, the Netherlands

**Table of Contents**

[**Supporting Texts** 3](#_Toc199698642)

[**Text S1.** Height and weight measurements in the Generation R Study. 3](#_Toc199698643)

[**Text S2.** Multivariate Imputation using Random Forest Modelling. 5](#_Toc199698644)

[**Text S3.** Inverse Probability Weight calculations. 10](#_Toc199698645)

[**Supporting Tables** 13](#_Toc199698646)

[**Table S1.** Non-imputed descriptive statistics. 13](#_Toc199698647)

[**Table S2.** Non-response analyses. 14](#_Toc199698648)

[**Table S3.** Correlation matrix of the continuous variables. 15](#_Toc199698649)

[**Table S4.** Independent associations of BMI per age in childhood with BMI and odds of overweight and obesity at 18 years, basic models. 16](#_Toc199698650)

[**Table S5.** Associations of BMI at different childhood ages with BMI and odds of overweight and obesity at 18 years. 16](#_Toc199698651)

[**Table S6.** Average N per group depicted in Figures 2A-D. 17](#_Toc199698652)

[**Table S7.** Exact proportions depicted in Figures 2A-D and their corresponding sex- and age-adjusted odds of overweight and obesity for each group. 17](#_Toc199698653)

[**Table S8**. Associations of BMI SDS change per age window and BMI outcomes at 18 years, basic models. 18](#_Toc199698654)

[**Table S9.** Associations of BMI SDS change per age window and BMI outcomes at 18 years, full models. 19](#_Toc199698655)

[**Supporting Figures** 20](#_Toc199698656)

[**Figure S1.** Flowchart of complete cases. 20](#_Toc199698657)

[**Figure S2.** Directed Acyclic Graph of investigated associations. 21](#_Toc199698658)

# **Supporting Texts**

## **Text S1. Details on variable measurements in the Generation R Study.**

**Height and weight**

In both the community health centers and research center, height was measured in a standing position to the nearest millimeter using a Harpenden stadiometer (Holtain Limited, Dyfed, UK). Children were asked to remove their shoes and any heavy clothing before measurement. Weight was measured to the nearest 0.1 kg using a mechanical personal scale (SECA, Almere, the Netherlands). To prevent any overlap in age groups, age cut-offs were applied for BMI measurements at each measurement time. For age 2, measurements conducted between 1.5 to 2.5 years were included (N = 0 measurement removals). For age 6, measurements conducted between 4.5 to 8.5 years were included (approximately N = 12 measurement exclusions per imputed dataset, rounded to nearest integer). For age 10, measurements conducted between 8.5 and 12.5 were included (approximately N = 2 measurement exclusions per imputed dataset, rounded to nearest integer). For age 14, measurements conducted between 12.5 and 16.0 years were included (approximately N = 4 measurement exclusions per imputed dataset, rounded to nearest integer).

We calculated BMI (weight(kg)/height(m)2), and sex- and age-adjusted-SDS for height and BMI based on World Health Organization (WHO) reference growth charts using R packages anthro and anthroplus. For those older than 19 years at outcome (n = 419), of which the majority (n=333) was below 20 years and the oldest 21.7 years, we calculated a proxy for their BMI-SDS as if they were exactly 19 years old. We used BMI-SDS cut-offs <1SD, ≥1SD and ≥2SD to categorize low/normal weight, overweight and obesity, respectively.

**Ethnicity variable**

Child ethnicity was defined according to the classification of Statistics Netherlands, based on birth place of parents obtained from questionnaires at intake. Children were defined as European if they were classified as Dutch or from another European country. Otherwise, they were defined as non-European.

**Puberty variables.**

The measured puberty factors consisted of self-reported Tanner stages for genital and pubic hair development for boys (N = 889), and breast and pubic hair development for girls (N = 1161) at age 13 years using a digital questionnaire in which they could select the Tanner stage image (1 to 5) that they believed to be most similar to their own body. At 13 and 17 years of age, female participants (N = 1120) were asked to report the age of first menstruation, if any.

**Text S2. Multivariate Imputation by Chained Equations (MICE) and Random Forest Modelling.**

Multivariate imputation by chained equations (MICE), also known as full conditional specification, is a common method of generating imputed values by drawing from estimated conditional distributions of each variable given by all the others.^(1)^ Imputation models must be appropriately specified for analyses based on imputed data to yield unbiased parameter estimates and associated standard errors. The default setting in implementations of MICE is for imputation models to include continuous variables as linear terms only with no interactions, but omission of important nonlinear terms may lead to biased results.^(1)^ Additionally, inclusion of highly correlated variables may cause problems due to multicollinearity. Random forest imputation is a method that was developed to overcome this and some of the other problems with ‘traditional’ MICE imputation implementations. Random forest uses bootstrap aggregation of multiple regression trees to reduce the risk of overfitting, and it combines the predictions from many trees to produce more accurate predictions, and has been widely used in other fields, including genetic epidemiology. Shah et al. developed and demonstrated a new version of MICE that implements random forest modelling. They modelled a complex survival analysis dataset, and found that parameter estimates were less biased using random forest MICE, and that confidence interval coverage was better, suggesting that this method can be useful for imputing complex epidemiologic datasets.^(1)^ However, it is still important to check whether your data is highly skewed or nonlinear, which in this study was visually inspected using density plots and histograms for all variables used in the imputation model.^(2)^

**Imputation conducted in this study**

In this study we conducted random forest imputation using the mice package (version 3.17.0) in R. The seed was set to 2024, we assumed MAR, and imputed the data using 30 iterations with 10 trees, and created a total of 30 imputed datasets. Imputation was conducted in the dataset prior to exclusion of participants, and included data on all participants who did not request removal of information and had data on a live-born child (N = 9746). This was done in order to try and capture as much information of the potential source population of our study sample. The variables used in the imputation model, along with their percentage missing and N, can be found in **Table SA.2**.

We additionally performed passive imputation based on the imputed variables. Passive imputation refers to the calculation of variables based on imputed variables, which takes place within the same iteration. Passive imputation was performed for total screen hours per day, total sleep in hours per day, sex- and gestational age-adjusted birthweight SDS, absolute BMI and the sex- and age-adjusted BMI Standard deviation scores (SDS) at each measurement age. Total screen hours per day was calculated based on the sum of the average amount of hours spent behind a computer, a television and a gaming device. Average sleep per day was calculated based on ((Average hours per day on weekdays*5) + (Average hours per day weekends*2))/7. Birth weight SDS was calculated based on birthweight references introduced by Nikklasson et al, with the following formula, adapted from this study, used for boys^(3)^:

$$\frac{\sqrt{BW} -(6.051836581 - 0.600816861 * GA + \left( 0.021243304* \left( \mathrm{GA}^{2} \right) \right)-(0.000220617*{GA}^{3})}{(-3.896362859 + 0.338487494 * GA - (0.009363082 * \mathrm{GA}^{2}) + 0.000085226 * (\mathrm{GA}^{3})}$$

And for girls:

$$\frac{\sqrt{BW}-(8.867334794 - (0.847020906 * GA )+ \left( 0.02826482* \left( \mathrm{GA}^{2} \right) \right)-(0.000286775*{GA}^{3})}{(-1.77611391+ 0.15567451 * GA - (0.00418448 * \mathrm{GA}^{2}) + 0.000036896 * (\mathrm{GA}^{3})}$$

Absolute BMI was calculated using the standard fomula weight in kg/(height in meters)^2^. The BMI SDS variables were calculated using the ‘anthro’ and ‘anthroplus’ (versions 1.0.1. and 1.0.0., respectively) packages developed by the WHO.^4,5^ Due to the nature of passive imputation, these calculated variables could not be used to impute the variables on which they are based. In other words, total sleep could not be used to impute sleep on weekdays or weekends, for example.

All imputed variables were checked and compared to the non-imputed dataset. No issues were observed in the directly imputed variables. However, for the passively imputed variables BMI and BMI SDS we observed some extreme outliers. In order to adjust for this in the analyses, the BMI SDS were winsorized to the minimum and maximum for that age as it was calculated the original, non-imputed dataset.

**Table SA.2.** Variables in imputation model and corresponding percentage missing (ascending order).

| **Variable** | **Percentage Missing** | **N missing** |
| --- | --- | --- |
| Maternal Age | 0.0 | 0 |
| Child Sex | 0.0 | 0 |
| Birthweight | 0.5 | 49 |
| Gestational age at birth | 0.8 | 78 |
| Parity | 3.6 | 350 |
| Child Ethnicity (European; Non-European) | 6.6 | 638 |
| Living situation (parent living together, yes/no) | 12.2 | 1180 |
| Maternal education (Higher, yes/no) | 12.3 | 1189 |
| Smoking during pregnancy (yes/no) | 15.5 | 1507 |
| Alcohol use during pregnancy (yes/no) | 21.9 | 2126 |
| Age biological father at intake | 22.9 | 2223 |
| Household income (>2200 euro’s/month, yes/no) | 25.7 | 2496 |
| Pre-pregnancy maternal BMI | 27.1 | 2628 |
| Child Age at measurement at 6 | 31.1 | 3011 |
| Height child at age 6 | 31.1 | 3014 |
| Weight child at age 6 | 31.1 | 3014 |
| Paternal BMI at intake | 35.2 | 3409 |
| Child age at measurement at 9 | 39.6 | 3839 |
| Weight child at age 9 | 41.2 | 3993 |
| Height child at age 9 | 41.2 | 3994 |
| Length at birth | 41.5 | 4026 |
| Child age at measurement at 24 months | 44.0 | 4266 |
| Weight child at 24 months | 44.1 | 4276 |
| Height child at 24 months | 44.9 | 4357 |
| Paternal education (Higher, yes/no) | 45.9 | 4448 |
| Age child at measurement at 13 | 49.2 | 4770 |
| Weight child at age 13 | 49.3 | 4779 |
| Height child at age 13 | 49.3 | 4780 |
| BMI SDS at age 17 (not imputed) | 62.6 | 6069 |
| Child age at measurement at 17 (not imputed) | 62.6 | 6069 |
| Height child at age 17 (not imputed) | 62.6 | 6069 |
| Weight child at age 17 (not imputed) | 62.6 | 6069 |
| Gaming hours/day | 62.6 | 6073 |
| Sleep hours/day weekdays | 62.6 | 6075 |
| Television hours/day | 62.7 | 6076 |
| Computer hours/day | 62.7 | 6080 |
| Sleep hours/day weekend days | 67.4 | 6533 |
| Note: This variable list does not contain the passively imputed variables. | | |

**References**

1. Shah AD, Bartlett JW, Carpenter J, Nicholas O, Hemingway H. Comparison of random forest and parametric imputation models for imputing missing data using MICE: a CALIBER study. Am J Epidemiol. 2014 Mar 15;179(6):764-74. doi: 10.1093/aje/kwt312. Epub 2014 Jan 12. PMID: 24589914; PMCID: PMC3939843.
2. Hong, S., Lynn, H.S. Accuracy of random-forest-based imputation of missing data in the presence of non-normality, non-linearity, and interaction. BMC Med Res Methodol 20, 199 (2020). <https://doi.org/10.1186/s12874-020-01080-1>
3. Niklasson A, Albertsson-Wikland K. Continuous growth reference from 24th week of gestation to 24 months by gender. *BMC Pediatr* 2008;**8:** 8.
4. anthro: Computation of the WHO Child Growth Standards. Version R package version 1.0.1. 2023. https://CRAN.R-project.org/package=anthro
5. anthroplus: Computation of the WHO 2007 References for School-Age Children and Adolescents (5 to 19 Years). Version R package version 1.0.0. 2024. https://CRAN.R-project.org/package=anthroplus

## **Text A3. Inverse Probability Weight calculations**.

**Creating Inverse Probability Weights for the Generation of Reference Centiles**

Inverse probability weighting is a method that creates weights to address potential biases. It was initially developed to address selection bias in surveys, as proposed by Horvitz and Thompson (1952), but has since then also been adapted and used to address confounding bias, also known as inverse probability of treatment weighting (IPTW).^(1)^ For this study, we applied inverse probability of censoring weighting (IPCW) in order to address selection bias introduced due to loss to follow-up in this cohort. This method can be applied in studies that have enough detailed information of participants recruited into the study available at baseline. The first step is to select variables at baseline that are related to response as well as the exposure outcome under study, in this case variables such as sex, gestational age or ethnicity. These variables are then used to estimate the probability of not being lost to follow-up. The weights are subsequently created by taking the inverse of those probabilities, resulting in weights that give more weight to individuals with a low probability of participation compared to individuals with a high probability of participation. This way participants with a lower probability of participation that nonetheless end up in the final analysis sample are given extra weight that helps them “represent” participants who were similar but that were actually lost to follow-up. We calculated the inverse probability weights similar to the method applied in a previous paper in the Generation R Study cohort.^(2)^

*Calculating the weights*

The weights were estimated by modeling the probability of being in the final analysis sample compared to being in the cohort at birth, conditional on the variables listed in the table below (**Table B.1**). Weights were calculated separately for each follow-up measurement, based on data of N = 9749 live born children in the cohort with parental consent within the Generation R Study. The probability of participation at the outcome measurement was calculated for each imputed dataset using the covariate balancing propensity score (CBPS) R package (version 0.23) and the CBPS function.^(3)^. After calculation, the inverse of those probabilities were taken as the weights used in the analyses.^(2)^ However, inverse probability weights may effect the SE’s, so in order to correct for this, we ran all multiple regression analyses with the R package survey (version 4.4-2), using the functions svydesign() and svyglm(). As these did not produce a standardized beta, we did so using a for-loop for each imputed dataset, and subsequently calculating the overall mean of each standardized beta and limits of the 95% confidence intervals across all imputed datasets. For beta’s, the standardized beta was calculated using:

$$\beta_{i}^{*}= \beta_{i}*\sigma_{xi}/\sigma_{y}$$

And for Odds Ratios this was done (if the exposure was not categorical) using:

$$e_{i}^{\beta^{*}}= e_{i}^{\beta}*\sigma_{xi}$$

This was all conducted in R Studio, using the version 4.4.1 version of R.

**Table A.3** **Covariates in the probability of follow up model.**

| **Variables in the model** | **Unit/Categories** |
| --- | --- |
| Sex | Boy, Girl |
| Gestational age at birth | Weeks |
| Ethnicity Child | European/Dutch and Non-European |
| Age mother at intake | Years |
| Maternal education | Higher education Yes and No |
| Paternal age at intake | Years |
| Maternal Ethnicity | European/Dutch and Non-European |
| Parents living together | Yes/No |
| Household income at intake | >2200 euros/month, <2200 euros/month |
| Maternal pre-pregnancy BMI | Kg/m^2^ |
| Paternal BMI at intake | Kg/m^2^ |
| Note: Intake took place either during pregnancy or shortly after giving birth. Abbreviations: BMI: Body Mass Index. | |

**References**

1. Horvitz, D. G., & Thompson, D. J. (1952). A Generalization of Sampling Without Replacement from a Finite Universe. Journal of the American Statistical Association, 47(260), 663–685. <https://doi.org/10.1080/01621459.1952.10483446>
2. Dijkzeul, A., Tiemeier, H., Muetzel, R. L., & Labrecque, J. A. (2024). Attention-deficit hyperactivity disorder symptoms and brain morphology: Addressing potential selection bias with inverse probability weighting. Human Brain Mapping, 45(5), e26562. https://doi.org/10.1002/hbm.26562
3. Imai K., & Ratkovic M. (2014). Covariate Balancing Propensity Score. Journal of the Royal Statistical Society Series B: Statistical Methodology, Volume 76(1), 243-263. <https://doi.org/10.1111/rssb.12027>

# **Supporting Tables**

## **Table S1.** Non-imputed descriptive statistics.

|  | **N = 3,528** |
| --- | --- |
| **Parental characteristics** | |
| Maternal age, years | 31.2 (4.9) |
| Multiparous, % (n) | 41.5 (1417) |
| Pre-pregnancy maternal BMI, kg/m^2^ | 22.4 (14.4; 50.2) |
| Father/partner BMI at intake, kg/m^2^ | 25.2 (3.4) |
| Maternal smoking during pregnancy, % (n) | 14.0 (432) |
| Household income≥2000 €/month,% (n) | 72.4 (2159) |
| Maternal higher education, % (n) | 54.3 (1776) |
| **Child characteristics** |  |
| Female, % (n) | 52.9 (1867) |
| European/Dutch, % (n) | 71.4 (2470) |
| **Child growth characteristics** |  |
| Gestational age at birth, weeks | 40.1 (25.9; 43.4) |
| Birth weight, kg | 3.45 (0.55) |
| **2 years** |  |
| Age, years | 2.1 (1.9; 2.4) |
| Body mass index, kg/m^2^ | 16.5 (1.4) |
| Body mass index, SDS |  |
| **6 years** |  |
| Age, years | 6.0 (4.8; 8.5) |
| Body mass index, kg/m^2^ | 15.8 (11.9; 28.3) |
| Body mass index, SDS |  |
| **10 years** |  |
| Age, years | 9.7 (8.5; 12.5) |
| Body mass index, kg/m^2^ | 16.8 (11.7; 35.4) |
| Body mass index, SDS |  |
| **14 years** |  |
| Age, years | 13.5 (12.6; 16.0) |
| Body mass index, kg/m^2^ | 19.0 (12.5; 43.9) |
| Body mass index, SDS |  |
| **18 years** |  |
| Age, years | 18.5 (16.2; 21.7) |
| Body mass index, kg/m^2^ | 22.0 (13.7; 49.5) |
| Body mass index, SDS |  |
| Underweight, % (n) |  |
| Healthy weight, % (n) |  |
| Overweight, % (n) | 15.1 (531) |
| Obesity, % (n) | 7.1 (252) |
| **Note:** Values are observed, non-imputed data and represent means (SD), medians (range) or valid % (n) unless otherwise stated. The mean and standard deviation are given for all normally distributed continuous variables, and the median and range for non-normally distributed variables. All child ages are reported as medians (range), regardless of their distribution. Abbreviations: BMI: Body Mass Index, SDS: standard deviation score (sex and age adjusted). | |

## **Table S2.** Non-response analyses.

|  | **Study population** | **Excluded** | | **P-value** |
| --- | --- | --- | --- | --- |
|  | Total N = 3528 | Total N = 6169 | |  |
|  | Summary statistic | Summary statistic | Valid N |  |
| **Base characteristics** | | | | |
| Female, % (n) | 52.9 (1867) | 47.5 (2922) | 6169 | <0.001** |
| European/Dutch, % (n) | 71.4 (2470) | 55.8 (3124) | 5601 | <0.001** |
| Age at outcome, years | 18.4 (0.6) | 18.5 (0.6) | 100 | 0.33 |
| Height at outcome, cm | 174.9 (9.6) | 174.7 (10.8) | 100 | 0.88 |
| **Pregnancy characteristics** |  |  |  |  |
| Household income≥2000 €/month,% (n) | 72.4 (2159) | 54.0 (2159) | 4217 | <0.001** |
| Maternal higher education, % (n) | 54.3 (1776) | 35.9 (1878) | 5237 | <0.001** |
| Pre-pregnancy maternal BMI, kg/m^2^ | 22.4 (18.1; 34.0) | 22.7 (17.8; 35.4) | 4405 | <0.001** |
| Father/partner BMI at intake, kg/m^2^ | 25.2 (3.4) | 25.4 (3.6) | 3712 | 0.04* |
| Sustained smoking during pregnancy, % (n) | 14.0 (432) | 20.5 (1046) | 5106 | <0.001** |
| Maternal age, years | 31.3 (4.9) | 29.2 (5.5) | 6169 | <0.001** |
| Parity, >0 % (n) | 41.5 (1417) | 47.0 (2788) | 5929 | <0.001** |
| **Birth characteristics** |  |  |  |  |
| Birthweight, kg | 3.45 (0.56) | 3.36 (0.59) | 6125 | <0.001** |
| Gestational age at birth, weeks | 40.1 (35.6; 42.3) | 39.7 (34.9; 42.3) | 6110 | <0.001** |
| **Childhood characteristics** |  |  |  |  |
| Sleep duration at 14 years, hours/day | 9.6 (0.8) | 9.5 (0.9) | 1048 | 0.38 |
| Screen-time, hours/day | 4.5 (2.2) | 4.5 (2.2) | 1184 | 0.24 |
| **Outcome** |  |  |  |  |
| BMI at 18 years, kg/m^2^ | 22.0 (17.4; 34.0) | 21.9 (17.6; 28.2) | 100 | 0.19 |
| Note: Values are observed. not imputed data and represent means (SD). medians (95% range) or valid % (n) unless otherwise stated. The mean and standard deviation are given for all normally distributed continuous variables. and the median and 95% range for non-normally distributed variables. P-values given are from independent t-tests. Mann-Whitney U tests or Chi-square tests for normally-distributed continuous outcomes. non-normally distributed continuous outcomes. and categorical outcomes. respectively. Abbreviations: BMI: Body Mass Index. *significant assuming a p-value<0.05. ** significant assuming a p-value <0.01 | | | | |

## **Table S3.** Correlation matrix of the continuous variables in the analyses.

|  | **Maternal age at intake** | **Pre-pregnancy BMI** | **Paternal BMI at intake** | **Parity** | **Birthweight SDS** | **BMI SDS at 2 years** | **BMI SDS at 6 years** | **BMI SDS at 10 years** | **BMI SDS at 14 years** | **Height SDS at 18** | **BMI SDS at 18** | |
| --- | --- | --- | --- | --- | --- | --- | --- | --- | --- | --- | --- | --- |
| **Maternal Age** | 1.00 | - | - | - | - | - | - | - | - | - | - | |
| **Pre-pregnancy BMI** | 0.00 | 1.00 | - | - | - | - | - | - | - | - | - | |
| **Paternal BMI at intake** | 0.05 | 0.22 | 1.00 | - | - | - | - | - | - | - | - | |
| **Parity** | 0.30 | 0.16 | 0.07 | 1.00 | - | - | - | - | - | - | - | |
| **Birthweight SDS** | 0.12 | 0.14 | 0.04 | 0.17 | 1.00 | - | - | - | - | - | - | |
| **BMI SDS at 2** | 0.00 | 0.15 | 0.10 | 0.05 | 0.24 | 1.00 | - | - | - | - | - | |
| **BMI SDS at 6** | -0.11 | 0.30 | 0.23 | 0.04 | 0.18 | 0.60 | 1.00 | - | - | - | - | |
| **BMI SDS at 10** | -0.11 | 0.34 | 0.30 | 0.06 | 0.12 | 0.43 | 0.81* | 1.00 | - | - | - | |
| **BMI SDS at 14** | -0.13 | 0.34 | 0.32 | 0.06 | 0.09 | 0.36 | 0.71 | 0.84* | 1.00 | - | - | |
| **Height SDS at 18** | 0.16 | -0.07 | -0.01 | -0.01 | 0.34 | 0.07 | -0.04 | -0.08 | -0.13 | 1.00 | - | |
| **BMI SDS at 18** | -0.10 | 0.32 | 0.29 | 0.03 | 0.10 | 0.29 | 0.59 | 0.69 | 0.78 | -0.07 | 1.00 | |
| Note: All the correlation values are the calculated Pearson’s R statistic. Abbreviations: BMI: Body Mass Index; SDS: Standard deviation score. *Correlation above 0.80. | | | | | | | | | | | |  |

|  | **Body Mass Index SDS** | | **Obesity/ Overweight presence** | |  |
| --- | --- | --- | --- | --- | --- |
|  | | Standardized β (95% CI) | | Standardized OR (95% CI) | |
| **Birth weight SDS^a^** | | 0.15 (0.l0; 0.19)** | | 1.24 (1.13; 1.37)** | |
| **BMI-SDS 2 years** | | 0.27 (0.22; 0.32)** | | 1.55 (1.37; 1.75)** | |
| **BMI-SDS 6 years** | | 0.51 (0.46; 0.56)** | | 2.71 (2.34; 3.14)** | |
| **BMI-SDS 10 years** | | 0.37 (0.31; 0.42)** | | 2.02 (1.76; 2.31)** | |
| **BMI-SDS 14 years** | | 0.34 (0.29; 0.40)** | | 1.92 (1.68; 2.21)** | |
| Note: All β’s and Odds Ratio’s (OR) and corresponding confidence intervals are pooled standardized estimates of 30 imputed datasets. All p-values were FDR-corrected. *p-value < 0.05. **p-value < 0.001. Abbreviations: BMI SDS: Body Mass Index Standard Deviation Score; CI: Confidence Interval; β: effect estimate. OR: Odds Ratio. ^a^Only for birthweight did we use the actual SDS as exposure instead of residuals. as there were no previous measurements to regress on. | | | | | |

## **Table S4.** Independent associations of BMI per age in childhood with BMI and odds of overweight and obesity at 18 years, basic models.

## **Table S5.** Associations of BMI at different childhood ages with BMI and odds of overweight and obesity at 18 years, full models.

|  |  | | **Adult Body Mass Index SDS** | **Adult Overweight/Obesity presence** |
| --- | --- | --- | --- | --- |
|  | | Standardized β (95% CI) | | Standardized OR (95% CI) |
| **Birth weight SDS^a^** | | 0.11 (0.06; 0.15)** | | 1.20 (1.08; 1.33)* |
| **BMI-SDS 2 years** | | 0.22 (0.18; 0.27)** | | 1.46 (1.29; 1.65)** |
| **BMI-SDS 6 years** | | 0.44 (0.39; 0.49)** | | 2.36 (2.04; 2.72)** |
| **BMI-SDS 10 years** | | 0.29 (0.23; 0.34)** | | 1.77 (1.54; 2.03)** |
| **BMI-SDS 14 years** | | 0.29 (0.24; 0.34)** | | 1.82 (1.59; 2.09)** |
| Note: All β’s and Odds Ratio’s (OR) and corresponding confidence intervals are pooled standardized estimates of 30 imputed datasets. All p-values were FDR-corrected. *p-value < 0.05, **p-value < 0.001. Abbreviations: BMI SDS: Body Mass Index Standard Deviation Score; CI: Confidence Interval; β: effect estimate, OR: Odds Ratio. ^a^Only for birthweight did we use the actual SDS as exposure instead of residuals, as there were no previous measurements to regress on. | | | | |

|  |  | | **Adult Body Mass Index SDS** | **Adult Overweight/Obesity presence** |
| --- | --- | --- | --- | --- |
|  | | Standardized β (95% CI) | | Standardized OR (95% CI) |
| **Birth weight SDS^a^** | | 0.14 (0.08; 0.20)** | | 1.30 ( (1.13; 1.49)* |
| **BMI-SDS 2 years** | | 0.23 (0.17; 0.28)** | | 1.49 (1.49; 1.77)** |
| **BMI-SDS 6 years** | | 0.43 (0.37; 0.48)** | | 2.35 (1.95; 2.84)** |
| **BMI-SDS 10 years** | | 0.30 (0.23; 0.37)** | | 1.76 (1.47; 2.12)** |
| **BMI-SDS 14 years** | | 0.36 (0.30; 0.41)** | | 2.13 (1.81; 2.51)** |
| Note: All β’s and Odds Ratio’s (OR) and corresponding confidence intervals are pooled standardized estimates of 30 imputed datasets. All p-values were FDR-corrected. *p-value < 0.05, **p-value < 0.001. Models were adjusted for child sex, ethnicity, household income, pre-pregnancy parental BMI, pregnancy smoking, maternal education, age at outcome, maternal age, parity, self-reported Tanner stages for pubic hair development and genital or breast development. Abbreviations: BMI SDS: Body Mass Index Standard Deviation Score; CI: Confidence Interval; β: effect estimate, OR: Odds Ratio. ^a^Only for birthweight did we use the actual SDS as exposure instead of residuals, as there were no previous measurements to regress on. | | | | |

## **Table S6.** Associations of BMI at different childhood ages with BMI and odds of overweight and obesity at 18 years, sensitivity analysis with puberty factors for both sexes N = 2,050 (full models).

## **Table S7.** Associations of BMI at different childhood ages with BMI and odds of overweight and obesity at 18 years, sensitivity analysis with puberty, sex-stratified (full models).

|  | **Adult Body Mass Index SDS** | | **Adult Overweight/Obesity presence** | |
| --- | --- | --- | --- | --- |
|  | Standardized β (95% CI) | | Standardized OR (95% CI) | |
|  | Girls  (N = 1120) | Boys  (N = 889) | Girls  (N = 1120) | Boys  (N = 889) |
| **Birth weight SDS^a^** | 0.17 (0.10; 0.25)** | 0.10 (0.00; 0.20)* | 1.47 ( (1.22; 1.77)* | 1.12 (0.88; 1.41) |
| **BMI-SDS 2 years** | 0.21 (0.13; 0.29)** | 0.24 (0.15; 0.33)** | 1.39 (1.10; 1.77)** | 1.51 (1.12; 2.03)* |
| **BMI-SDS 6 years** | 0.44 (0.37; 0.52)** | 0.41 (0.32; 0.50)** | 2.44 (1.87; 3.19)** | 2.20 (1.62; 2.98)** |
| **BMI-SDS 10 years** | 0.28 (0.19; 0.37)** | 0.31 (0.21; 0.41)** | 1.85 (1.45; 2.36)** | 1.70 (1.29; 2.23)** |
| **BMI-SDS 14 years** | 0.24 (0.17; 0.31)** | 0.43 (0.35; 0.52)** | 1.82 (1.47; 2.26)** | 2.32 (1.80 2.97)** |
| Note: All β’s and Odds Ratio’s (OR) and corresponding confidence intervals are pooled standardized estimates of 30 imputed datasets. Models were stratified by sex and adjusted for child sex, ethnicity, household income, pre-pregnancy parental BMI, pregnancy smoking, maternal education, age at outcome, maternal age, parity, self-reported Tanner stages for pubic hair development and genital or breast development, as well as first age of menstruation for girls. All p-values were FDR-corrected. *p-value < 0.05, **p-value < 0.001. Abbreviations: BMI SDS: Body Mass Index Standard Deviation Score; CI: Confidence Interval; β: effect estimate, OR: Odds Ratio. ^a^Only for birthweight did we use the actual SDS as exposure instead of residuals, as there were no previous measurements to regress on. | | | | |

## **Table S8.** Associations of BMI at different childhood ages with BMI and odds of overweight and obesity at 18 years, complete case analysis N = 1,971 (full models).

|  |  | | **Adult Body Mass Index SDS** | **Adult Overweight/Obesity presence** |
| --- | --- | --- | --- | --- |
|  | | Standardized β (95% CI) | | Standardized OR (95% CI) |
| **Birth weight SDS^a^** | | 0.10 (0.04; 0.16)** | | 1.13 (0.98; 1.31) |
| **BMI-SDS 2 years** | | 0.27 (0.21; 0.33)** | | 1.58 (1.37; 1.83)** |
| **BMI-SDS 6 years** | | 0.49 (0.44; 0.55)** | | 2.69 (2.26; 3.20)** |
| **BMI-SDS 10 years** | | 0.32 (0.26; 0.38)** | | 1.84 (1.56; 2.17)** |
| **BMI-SDS 14 years** | | 0.36 (0.31; 0.42)** | | 2.13 (1.82; 2.50)** |
| Note: All β’s and Odds Ratio’s (OR) and corresponding confidence intervals are pooled standardized estimates of 30 imputed datasets. All p-values were FDR-corrected. *p-value < 0.05, **p-value < 0.001. Models were adjusted for child sex, ethnicity, household income, pre-pregnancy parental BMI, pregnancy smoking, maternal education, age at outcome, maternal age and parity. Abbreviations: BMI SDS: Body Mass Index Standard Deviation Score; CI: Confidence Interval; β: effect estimate, OR: Odds Ratio. ^a^Only for birthweight did we use the actual SDS as exposure instead of residuals, as there were no previous measurements to regress on. | | | | |

## **Table S9.** N per group depicted in Figures 2A-D.

|  | **Birth to 2 years** | **2 to 6 years** | **6 to 10 years** | **10 to 14 years** |
| --- | --- | --- | --- | --- |
| **Group** | N* | N* | N* | N* |
| **Low to Low** | 496 | 689 | 837 | 855 |
| **Low to Average** | 371 | 315 | 268 | 263 |
| **Low to High** | 282 | 152 | 70 | 47 |
| **Average to Low** | 373 | 332 | 286 | 274 |
| **Average to Average** | 381 | 471 | 594 | 636 |
| **Average to High** | 397 | 336 | 266 | 252 |
| **High to Low** | 290 | 146 | 39 | 37 |
| **High to Average** | 392 | 351 | 297 | 252 |
| **High to High** | 506 | 682 | 842 | 889 |
| **Note:** *The N is an average N across all the imputed datasets. Some weights were imputed. resulting in slightly different distributions in weight per imputed dataset. The average N was calculated and rounded to the nearest integer. | | | | |

## **Table S10.** Exact proportions depicted in Figures 2A-D and their corresponding sex- and age-adjusted odds of overweight and obesity for each group.

|  | **Birth to 2 years** | | **2 to 6 years** | | **6 to 10 years** | | **10 to 14 years** | |
| --- | --- | --- | --- | --- | --- | --- | --- | --- |
| **Group** | % (SE) | OR (95% CI) | % (SE) | OR (95% CI) | % (SE) | OR (95% CI) | % (SE) | OR (95% CI) |
| Stable Low | 13.9 (1.6) | 0.76 (0.48; 1.20) | 6.6 (0.9) | 0.43 (0.27; 0.67)* | 2.7 (0.6) | 0.22 (0.13; 0.39)* | 1.2 (0.3) | 0.14 (0.06; 0.33)* |
| Low to Average | 20.6 (2.1) | 1.12 (0.72; 1.76) | 17.0 (2.1) | 1.33 (0.80; 2.23) | 11.4 (1.9) | 0.94 (0.57; 1.56) | 5.7 (1.4) | 0.66 (0.34; 1.29) |
| Low to High | 34.0 (2.8) | 2.25 (1.46; 3.47)* | 45.8 (4.1) | 4.81 (2.83; 8.20)* | 38.0 (5.7) | 5.08 (2.57; 10.06)* | 24.9 (6.2) | 4.01 (1.64; 9.80)* |
| Average to Low | 15.5 (1.9) | 0.83 (0.50; 1.37) | 6.5 (1.3) | 0.40 (0.20; 0.78)* | 3.7 (1.1) | 0.31 (0.14; 0.70)* | 2.9 (1.0) | 0.37 (0.13; 1.04) |
| Stable Average | 18.3 (2.0) | Reference | 14.6 (1.6) | Reference | 11.4 (1.3) | Reference | 8.2 (1.1) | Reference |
| Average to High | 29.6 (2.3) | 1.89 (1.25; 2.85)* | 39.1 (2.7) | 3.68 (2.42; 5.61)* | 36.1 (2.4) | 4.31 (2.85; 6.52)* | 33.2 (3.0) | 4.94 (3.09; 7.90)* |
| High to Low | 14.4 (2.1) | 0.79 (0.47; 1.33) | 8.5 (0.4) | 0.47 (0.18; 1.24) | 8.7 (0.1) | 0.86 (0.13; 5.64) | 13.3 (1.0) | 1.99 (0.50; 7.82) |
| High to Average | 19.5 (2.0) | 1.05 (0.67; 1.63) | 14.3 (1.9) | 0.96 (0.59; 1.57) | 14.9 (2.1) | 1.25 (0.76; 2.06) | 14.2 (2.2) | 1.63 (0.91; 2.92) |
| Stable High | 33.1 (2.1) | 2.19 (1.49; 3.21)* | 46.4 (1.9) | 5.12 (3.54; 7.40)* | 56.2 (1.7) | 9.89 (7.12; 13.72)* | 62.5 (1.6) | 19.26 (13.32; 27.85)* |
| **Note:** The proportion of cases of overweight or obesity within each group are given as percentages (SE). The Standard Error (SE) of each percentage is given. as these proportions are pooled proportions based on 30 imputed datasets. As some weights were imputed for some ages, the proportion of overweight and/or obesity might differ slightly between imputations. the variation of which is indicated by the SE. *FDR-adjusted p-value <0.05**.** The reported Odds Ratio’s (ORs) were adjusted for sex at birth, age at outcome and the inverse probability weights, with the stable average group as the reference category of the independent variable. | | | | | | | | |

## **Table S11**. Associations of BMI SDS change per age window and BMI outcomes at 18 years, basic models.

|  | | **Body Mass Index SDS** | **Obesity/ Overweight presence** |  |
| --- | --- | --- | --- | --- |
|  | | Standardized β (95% CI) | Standardized OR (95% CI) |  |
| **BMI-SDS Δ (cont.)** | |  |  |  |
| Birth to 2 years | | 0.15 (0.09; 0.20)** | 1.27 (1.13; 1.43)** |  |
| 2 to 6 years | | 0.28 (0.23; 0.33)** | 1.65 (1.46; 1.87)** |  |
| 6 to 10 years | | 0.23 (0.17; 0.30)** | 1.55 (1.35; 1.77)** |  |
| 10 to 14 years | | 0.15 (0.09; 0.21)** | 1.30 (1.16; 1.46)** |  |
| 14 to 18 years | | 0.40 (0.35; 0.44)** | 2.07 (1.85; 2.32)** |  |
| **BMI-SDS Δ (cat.)** | |  |  |  |
| Birth - 2 years | Decelerated | -0.07 (-0.22; 0.08) | 1.05 (0.75; 1.48)* |  |
|  | Stable | Reference | Reference |  |
|  | Accelerated | 0.21 (0.11; 0.31)** | 1.54 (1.22; 1.94)** |  |
| 2 – 6 years | Decelerated | -0.23 (-0.34; -0.13)** | 0.62 (0.47; 0.83)** |  |
|  | Stable | Reference | Reference |  |
|  | Accelerated | 0.56 (0.42; 0.70)** | 2.57 (1.99; 3.32)** |  |
| 6 – 10 years | Decelerated | -0.26 (-0.40; -0.13)** | 0.65 (0.46; 0.91)* |  |
|  | Stable | Reference | Reference |  |
|  | Accelerated | 0.51 (0.37; 0.66)** | 2.58 (2.00; 3.33)** |  |
| 10 – 14 years | Decelerated | -0.29 (-0.40; -0.17)** | 0.59 (0.45; 0.77)** |  |
|  | Stable | Reference | Reference |  |
|  | Accelerated | 0.25 (0.07; 0.43)* | 1.55 (1.13; 2.14)* |  |
| Note: All β’s and Odds Ratio’s (OR) are standardized. pooled estimates taken from 30 imputed datasets. *p-value <0.05; ** p-value <0.001. Models were adjusted for age at outcome, child sex and ethnicity. Abbreviations: BMI: Body Mass Index; SDS: Standard Deviation Score; CI: Confidence Interval; cont.: continuous; cat.: categorical. | | | | |

## **Table S12.** Associations of BMI SDS change per age window and BMI outcomes at 18 years, full models.

|  | | **Body Mass Index SDS** | **Overweight/Obesity presence** |  |
| --- | --- | --- | --- | --- |
|  | | Standardized β (95% CI) | Standardized OR (95% CI) |  |
| **BMI SDS Δ (cont.)** | |  |  |  |
| Birth to 2 years | | 0.13 (0.09; 0.18)** | 1.24 (1.11; 1.40)** |  |
| 2 to 6 years | | 0.23 (0.18; 0.28)** | 1.53 ((1.36; 1.74)** |  |
| 6 to 10 years | | 0.18 (0.12; 0.24)** | 1.41 (1.23; 1.62)** |  |
| 10 to 14 years | | 0.13 (0.08; 0.18)** | 1.30 (1.16; 1.46)** |  |
| **BMI SDS Δ (cat.)** | |  |  |  |
| Birth - 2 years | Decelerated | -0.08 (-0.20; 0.05) | 1.06 (0.74; 1.50) |  |
|  | Stable | Reference | Reference |  |
|  | Accelerated | 0.18 (0.08; 0.27)** | 1.49 (1.15; 1.93)* |  |
| 2 – 6 years | Decelerated | -0.18 (-0.28; -0.08)** | 0.67 (0.49; 0.90)* |  |
|  | Stable | Reference | Reference |  |
|  | Accelerated | 0.45 (0.32; 0.58)** | 2.26 (1.73; 2.96)** |  |
| 6 – 10 years | Decelerated | -0.20 (-0.32; -0.09)** | 0.68 (0.48; 0.97)* |  |
|  | Stable | Reference | Reference |  |
|  | Accelerated | 0.39 (0.25; 0.53)** | 2.14 (1.62; 2.83)** |  |
| 10 – 14 years | Decelerated | -0.29 (-0.40; -0.19)** | 0.54 (0.40; 0.72)** |  |
|  | Stable | Reference | Reference |  |
|  | Accelerated | 0.16 (0.00; 0.32) | 1.42 (0.99; 2.03) |  |
| Note: All β’s and Odds Ratio’s (OR) are standardized, pooled estimates taken from 30 imputed datasets. * p-value <0.05, ** p-value <0.001. Models were adjusted for child sex, ethnicity, household income, pre-pregnancy parental BMI, pregnancy smoking, maternal education, age at outcome, maternal age, and parity. Abbreviations: BMI: Body Mass Index; SDS: Standard Deviation Score; CI: Confidence Interval; cont.: continuous; cat.: categorical. | | | | |

## **Table S13.** Associations of BMI SDS change per age window and BMI outcomes at 18 years, sensitivity analyses adjusting for puberty factors for both sexes, full models.

|  | | **Body Mass Index SDS** | **Overweight/Obesity presence** |  |
| --- | --- | --- | --- | --- |
|  | | Standardized β (95% CI) | Standardized OR (95% CI) |  |
| **BMI SDS Δ (cont.)** | |  |  |  |
| Birth to 2 years | | 0.12 (0.06; 0.18)** | 1.21 (1.03; 1.42)* |  |
| 2 to 6 years | | 0.19 (0.13; 0.26)** | 1.47 (1.23; 1.76)** |  |
| 6 to 10 years | | 0.19 (0.11; 0.27)** | 1.42 (1.19; 1.69)** |  |
| 10 to 14 years | | 0.17 (0.11; 0.23)** | 1.42 (1.23; 1.64)** |  |
| **BMI SDS Δ (cat.)** | |  |  |  |
| Birth - 2 years | Decelerated | -0.01 (-0.18; 0.16) | 1.33 (0.84; 2.09) |  |
|  | Stable | Reference | Reference |  |
|  | Accelerated | 0.18 (0.06; 0.31)** | 1.55 (1.08; 2.22)* |  |
| 2 – 6 years | Decelerated | -0.20 (-0.32; -0.07)** | 0.64 (0.43; 0.96)* |  |
|  | Stable | Reference | Reference |  |
|  | Accelerated | 0.35 (0.17; 0.53)** | 2.13 (1.46; 3.12)** |  |
| 6 – 10 years | Decelerated | -0.18 (-0.34; -0.02)* | 0.72 (0.45; 1.16) |  |
|  | Stable | Reference | Reference |  |
|  | Accelerated | 0.39 (0.21; 0.57)** | 2.22 (1.52; 3.25)** |  |
| 10 – 14 years | Decelerated | -0.40 (-0.52; -0.28)** | 0.35 (0.23; 0.53)** |  |
|  | Stable | Reference | Reference |  |
|  | Accelerated | 0.18 (-0.03; 0.40) | 1.33 (0.84; 2.09) |  |
| Note: All β’s and Odds Ratio’s (OR) are standardized, pooled estimates taken from 30 imputed datasets. * p-value <0.05, ** p-value <0.001. Models were adjusted for child sex, ethnicity, household income, pre-pregnancy parental BMI, pregnancy smoking, maternal education, age at outcome, maternal age, parity, and self-reported Tanner stages for pubic hair and genital or breast development. Abbreviations: BMI: Body Mass Index; SDS: Standard Deviation Score; CI: Confidence Interval; cont.: continuous; cat.: categorical. | | | | |

## **Table S14.** Associations of BMI SDS change per age window and BMI outcomes at 18 years, full models.

|  | | **Body Mass Index SDS** | | **Overweight/Obesity presence** | |
| --- | --- | --- | --- | --- | --- |
|  | | Standardized β (95% CI) | | Standardized OR (95% CI) | |
| **BMI SDS Δ (cont.)** | | **Girls** | **Boys** | **Girls** | **Boys** |
| Birth to 2 years | | 0.07 (-0.01; 0.15) | 0.16 (0.06; 0.25)** | 1.06 (0.85; 1.31) | 1.34 (1.01; 1.79)* |
| 2 to 6 years | | 0.20 (0.11; 0.29)** | 0.18 (0.08; 0.28)** | 1.51 (1.18; 1.92)** | 1.42 (1.06; 1.90)* |
| 6 to 10 years | | 0.16 (0.06; 0.26)** | 0.21 (0.10; 0.32)** | 1.44 (1.15; 1.80)** | 1.42 (1.10; 1.85)* |
| 10 to 14 years | | 0.06 (0.00; 0.13) | 0.25 (0.16; 0.34)** | 1.22 (1.02; 1.46)* | 1.59 (1.26; 2.01)** |
| **BMI SDS Δ (cat.)** | |  |  |  |  |
| Birth - 2 years | Decelerated | 0.10 (-0.12; 0.31) | -0.14 (-0.41; 0.13) | 1.80 (1.01; 3.22) | 0.86 (0.35; 2.08) |
|  | Stable | Reference | Reference | Reference | Reference |
|  | Accelerated | 0.14 (-0.01; 0.29) | 0.21 (0.02; 0.39) | 1.38 (0.85; 2.22) | 1.59 (0.91; 2.78) |
| 2 – 6 years | Decelerated | -0.17 (-0.32; -0.02) | -0.24 (-0.45; -0.03) | 0.66 (0.40; 1.09) | 0.54 (0.26; 1.10) |
|  | Stable | Reference | Reference | Reference | Reference |
|  | Accelerated | 0.41 (0.18; 0.65)** | 0.26 (-0.01; 0.53) | 2.23 (1.33; 3.74)* | 1.83 (0.99; 3.38) |
| 6 – 10 years | Decelerated | -0.19 (-0.40; 0.03) | -0.15 (-0.37; 0.06) | 0.68 (0.35; 1.32) | 0.78 (0.40; 1.52) |
|  | Stable | Reference | Reference | Reference | Reference |
|  | Accelerated | 0.24 (-0.01; 0.50) | 0.52 (0.26; 0.77)** | 1.93 (1.11; 3.34)* | 2.75 (1.58; 4.77)** |
| 10 – 14 years | Decelerated | -0.31 (-0.47; -0.15)** | -0.43 (-0.60; -0.26)** | 0.31 (0.16; 0.58)** | 0.38 (0.21; 0.69)* |
|  | Stable | Reference | Reference | Reference | Reference |
|  | Accelerated | -0.01 (-0.25; 0.23) | 0.41 (-0.02; 0.85) | 1.04 (0.58; 1.87) | 1.55 (0.66; 3.62) |
| Note: All β’s and Odds Ratio’s (OR) are standardized, pooled estimates taken from 30 imputed datasets. * p-value <0.05, ** p-value <0.001. Models were adjusted for child sex, ethnicity, household income, pre-pregnancy parental BMI, pregnancy smoking, maternal education, age at outcome, maternal age, parity, and self-reported Tanner stages for pubic hair and genital or breast development, as well as age of first menstruation for girls. Abbreviations: BMI: Body Mass Index; SDS: Standard Deviation Score; CI: Confidence Interval; cont.: continuous; cat.: categorical. | | | | | |

**Table S15.** Sensitivity analysis with puberty factors for both sexes: Change in BMI SDS per age window stratified for starting BMI and odds of overweight, including Obesity, at 18 years.

|  | **Overweight, including Obesity, at age 18 years**  Odds Ratio (95% Confidence Interval) | | | | | | | | | | | | | |
| --- | --- | --- | --- | --- | --- | --- | --- | --- | --- | --- | --- | --- | --- | --- |
|  | **Birth to 2 years** | | | **2 to 6 years** | | | **6 to 10 years** | | | **10 to 14 years** | | | | |
| **Baseline BMI** | **Decelerated** | **Stable** | **Accelerated** | **Decelerated** | **Stable** | **Accelerated** | **Decelerated** | **Stable** | **Accelerated** | **Decelerated** | **Stable** | **Accelerated** | |  |
| **Lowest**  **Tertile** | N.A.  (no cases)^a^ | 0.54  (0.23; 1.27) | 1.32  (0.78; 2.22) | 0.39  (0.12; 1.29) | 0.46  (0.26; 0.83)* | 1.82  (1.04; 3.20) | N.A.  (no cases)^a^ | 0.29  (0.16; 0.52)* | 1.74  (0.89; 3.40) | N.A.  (no cases)^a^ | 0.34  (0.17; 0.65)* | 1.21  (0.55; 2.68) | |  |
| **Middle**  **Tertile** | 1.28  (0.48; 3.39) | Reference | 1.78  (0.99; 3.21) | 0.26  (0.09; 0.77)* | Reference | 4.23  (1.98; 9.01)* | 0.33  (0.11; 0.92) | Reference | 3.38  (1.77; 6.45)* | 0.24  (0.07; 0.83)* | Reference | 4.19  (1.50; 11.67)* | |  |
| **Highest**  **Tertile** | 1.62  (0.90; 2.90) | 1.60  (0.91; 2.82) | 3.03  (1.53; 6.01)* | 1.15  (0.68; 1.95) | 3.39  (2.10; 5.47)**^*^** | 12.58  (3.75; 42.21)* | 2.14  (1.19; 3.85)* | 4.40  (2.99; 6.49)* | 8.94  (4.34; 18.41)* | 2.04  (1.18; 3.51)* | 11.47  (7.77; 16.94)* | N.A.  (no control)^a^ | |  |
| Note: All Odds Ratio’s (OR) and 95% Confidence Intervals are pooled estimates taken from 30 imputed datasets. The models were adjusted for age difference, child sex, maternal education and age, parental pre-pregnancy BMI, parity, household income, pregnancy smoking, and self-reported Tanner stages for pubic hair and genital or breast development. Decelerated and accelerated growth was defined as a BMI change of <-0.67 SDS and >0.67 SDS, respectively. The respective N for each group is given with each OR, and was based on the average N per group across all 31 imputed datasets, rounded to the nearest integer. *FDR-adjusted p < 0.05; ^a^This was defined as less than 1 case on average per imputed dataset. | | | | | | | | | | | | |  |  |

## **Table S16.** Associations of BMI SDS change per age window and BMI outcomes at 18 years, complete case analysis N = 1,971 (full models).

|  | | **Body Mass Index SDS** | **Overweight/Obesity presence** |  |
| --- | --- | --- | --- | --- |
|  | | Standardized β (95% CI) | Standardized OR (95% CI) |  |
| **BMI SDS Δ (cont.)** | |  |  |  |
| Birth to 2 years | | 0.18 (0.12; 0.23)** | 1.37 (1.18; 1.58)** |  |
| 2 to 6 years | | 0.26 (0.20; 0.32)** | 1.66 ((1.45; 1.91)** |  |
| 6 to 10 years | | 0.20 (0.14; 0.26)** | 1.45 (1.25; 1.68)** |  |
| 10 to 14 years | | 0.17 (0.13; 0.22)** | 1.43 (1.25; 1.63)** |  |
| **BMI SDS Δ (cat.)** | |  |  |  |
| Birth - 2 years | Decelerated | -0.16 (-0.31; -0.01)* | 0.93 (0.61; 1.40) |  |
|  | Stable | Reference | Reference |  |
|  | Accelerated | 0.15 (0.05; 0.26)** | 1.49 (1.11; 1.99)* |  |
| 2 – 6 years | Decelerated | -0.19 (-0.29; -0.09)** | 0.58 (0.41; 0.83)** |  |
|  | Stable | Reference | Reference |  |
|  | Accelerated | 0.57 (0.40; 0.73)** | 2.75 (1.96; 3.86)** |  |
| 6 – 10 years | Decelerated | -0.24 (-0.39; -0.09)* | 0.63 (0.39; 0.99) |  |
|  | Stable | Reference | Reference |  |
|  | Accelerated | 0.45 (0.28; 0.61)** | 2.25 (1.56; 3.26)** |  |
| 10 – 14 years | Decelerated | -0.31 (-0.42; -0.19)** | 0.46 (0.32; 0.68)** |  |
|  | Stable | Reference | Reference |  |
|  | Accelerated | 0.28 (0.09; 0.47) | 1.57 (1.01; 2.46) |  |
| Note: All β’s and Odds Ratio’s (OR) are standardized, pooled estimates taken from 30 imputed datasets. * p-value <0.05, ** p-value <0.001. Abbreviations: BMI: Body Mass Index; SDS: Standard Deviation Score; CI: Confidence Interval; cont.: continuous; cat.: categorical. | | | | |

# **Supporting Figures**

## **Figure S1.** Flowchart of complete cases.

**Live-born children**

Live born children with parental consent (n = 9749)

**Excluded: n = 247**

Twins (n= 243)

Removal of data at request of participant (n=3)

No information on sex (n=4)

**Live-born, singleton children with information on sex (n = 9502)**

**Excluded: n = 43**

No birthweight and gestational age data (n = 9)

Died shortly after birth (within 2 years) (n = 34)

**Excluded: n = 5931** *(60.8%)*

No known BMI at 18 years

**Total study population n = 3528**

BMI at 2 years n = 2234

BMI at 6 years n = 3236

BMI at 10 years n = 3101

BMI at 14 years n = 3050

**Live-born, singleton children with information on sex and birthweight, and who were alive for 1^st^ follow-up (n = 9459)**

**Note:** Removal of information of participant was done at the request of participants. This could be done at any time during their participation, or after their withdrawal. The 1^st^ follow up was at around the age of 6, followed by a second follow-up around 10 years old, a third follow-up at age 14 and their most recent follow-up at 18 years.

## **Figure S2.** Directed Acyclic Graph of investigated associations.

BMI change in childhood (birth to 14 years)

BMI at 18 years

Childhood

Self-reported Tanner stages

Age of first menstruation

Birth

Sex

Ethnicity

Pregnancy

Household income at intake

Maternal education at intake

Pre-pregnancy BMI Father/Partner BMI at intake

Smoking during pregnancy

Maternal age at intake

Parity

Age@18 BMI measurement

Age interval of change

**Note:** The above figure shows all potential variables that were measured in the Generation R Study that were considered as either confounding variables or precision variables. Abbreviations: BMI: Body Mass Index.
